# Supplementary material for: Type II topoisomerase substrate geometry revealed through combined experiment and computation
Source: bioRxiv. 2026 Sep 8:2026.09.04.749274. Preprint. [Version 1] doi: 10.64898/2026.09.04.749274 (PMC13622454; doi:10.64898/2026.09.04.749274)
Supplement: Supplementary file 1 [file NIHPP2026.09.04.749274V1-supplement-1.pdf]

bioRxiv preprint doi: https://doi.org/10.64898/2026.09.04.749274; this version posted September 8, 2026. The copyright holder for this preprint (which was not certified by peer review) is the author/funder. This article is a US Government work. It is not subject to copyright under 17 USC 105 and is also made available for use under a CC0 license.

Supporting Information for

Type II topoisomerase substrate geometry revealed through combined experiment and computation.

Mihirkumar N. Prajapati[1,2], Yeonee Seol[2], Jonathan Silver[2], Parth R. Desai[1,2,3], Siddhartha Das[1], Keir C. Neuman[2, *]

[1] Department of Mechanical Engineering, University of Maryland, College Park, MD 20742, USA

[2] Laboratory of Single Molecule Biophysics, National Heart, Lung, and Blood Institute, National Institutes of Health, Bethesda, MD 20892, USA

[3] Department of Biochemistry and Biophysics, Stockholm University, Stockholm, Sweden

* Corresponding author: Dr. Keir C. Neuman

Email: neumankc@nih.gov; Tel: +1 301 480 4076; Fax: +1 301 402 3404

This PDF file includes:

Supporting text

Figures S1 to S11

Tables S1 to S2

Legends for Movie S1

SI References

Other supporting materials for this manuscript include the following:

Movie S1

bioRxiv preprint doi: https://doi.org/10.64898/2026.09.04.749274; this version posted September 8, 2026. The copyright holder for this preprint (which was not certified by peer review) is the author/funder. This article is a US Government work. It is not subject to copyright under 17 USC 105 and is also made available for use under a CC0 license.

S1. Topo IV unlinking rate as a function of bead rotation

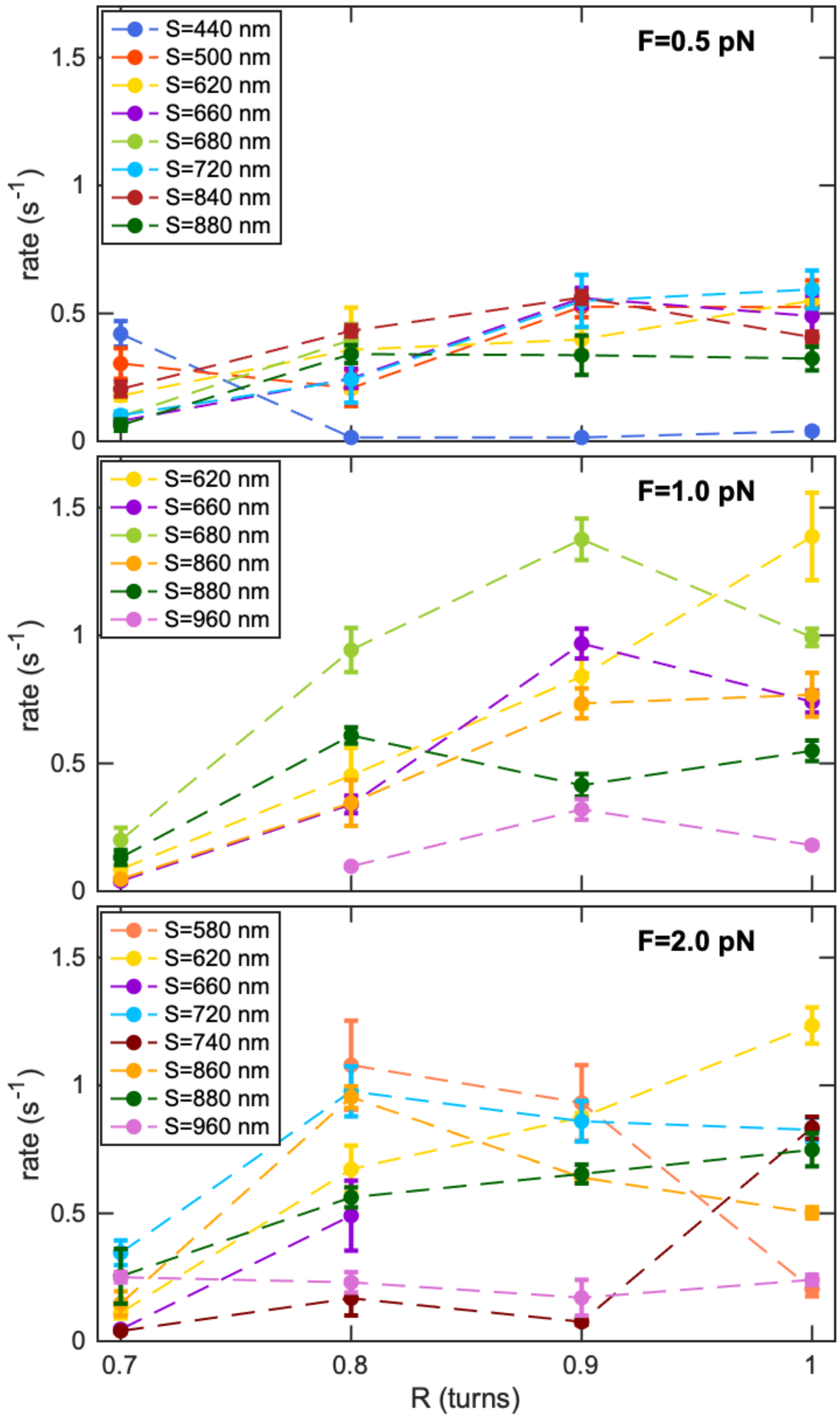

Figure S1: Topo IV unlinking rate as a function of bead rotation, R, for 12 different segment spacings (S) and three different forces (F). The unlinking rates measured are color-coded based on the segment spacing (S = 440 nm: Royal blue; 500 nm: Orange-red; 580 nm: Coral; 620nm: Gold; 660 nm: Dark violet; 680 nm: Yellow-green; 720 nm: Deep sky blue; 740 nm: Maroon; 840 nm: Firebrick; 860 nm: Orange; 880 nm: Dark green; 960 nm: Orchid) for three different forces

bioRxiv preprint doi: https://doi.org/10.64898/2026.09.04.749274; this version posted September 8, 2026. The copyright holder for this preprint (which was not certified by peer review) is the author/funder. This article is a US Government work. It is not subject to copyright under 17 USC 105 and is also made available for use under a CC0 license.

(F=0.5: Top panel; 1 pN: Middle panel; 2 pN: Bottom panel). Dashed lines between points are included to guide the eye. Error bars correspond to the standard error of the mean.

bioRxiv preprint doi: https://doi.org/10.64898/2026.09.04.749274; this version posted September 8, 2026. The copyright holder for this preprint (which was not certified by peer review) is the author/funder. This article is a US Government work. It is not subject to copyright under 17 USC 105 and is also made available for use under a CC0 license.

S2. Schematic of DNA crossing and parameter definitions

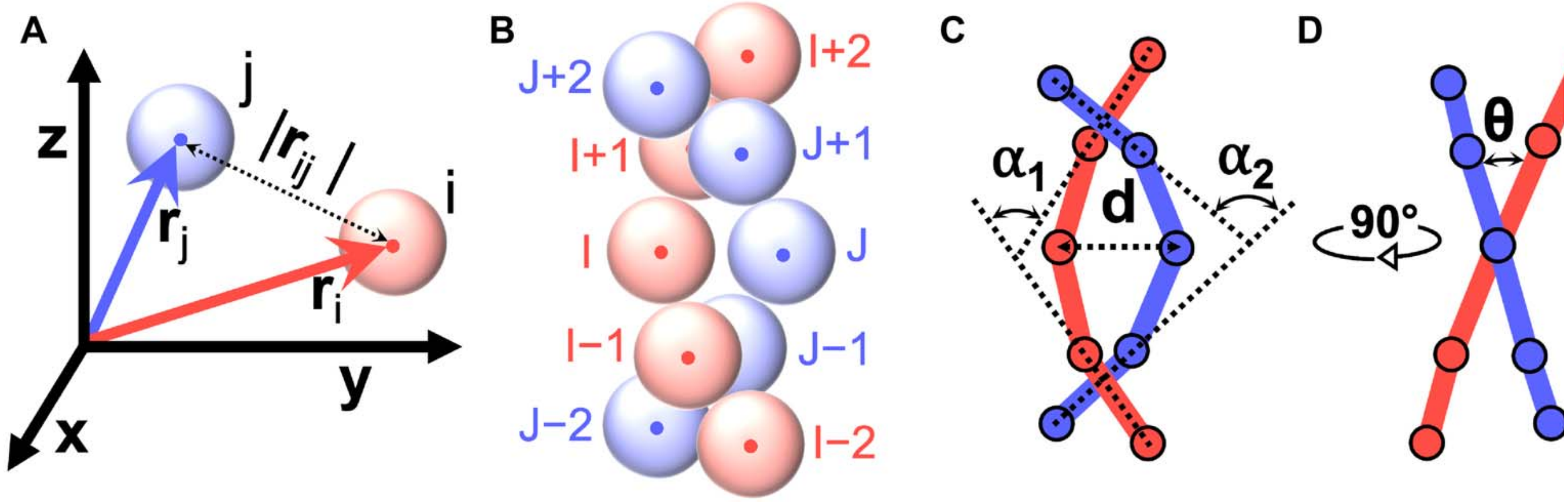

Figure S2: Schematic of DNA crossing and parameter definitions. **A.** Representative position vectors of monomer beads. **B.** Schematic of a crossing consisting of 5-monomer-bead segments from each dsDNA. **C.** Simplified schematic of a crossing with the ball-and-stick model (representing particle-and-bond) for easier visualization. The juxtaposition distance (*d*) is defined as the distance at the closest approach, i.e., central beads of the pair. The bend angles (α_1, α_2) are defined as the angle between the first and last bonds of the respective segments. **D.** Side view of the previous schematic. The crossing angle (θ) is defined as the angle between the planes defined by the two segments.

bioRxiv preprint doi: https://doi.org/10.64898/2026.09.04.749274; this version posted September 8, 2026. The copyright holder for this preprint (which was not certified by peer review) is the author/funder. This article is a US Government work. It is not subject to copyright under 17 USC 105 and is also made available for use under a CC0 license.

S3. Similarity of the probability distributions of α_1 and α_2

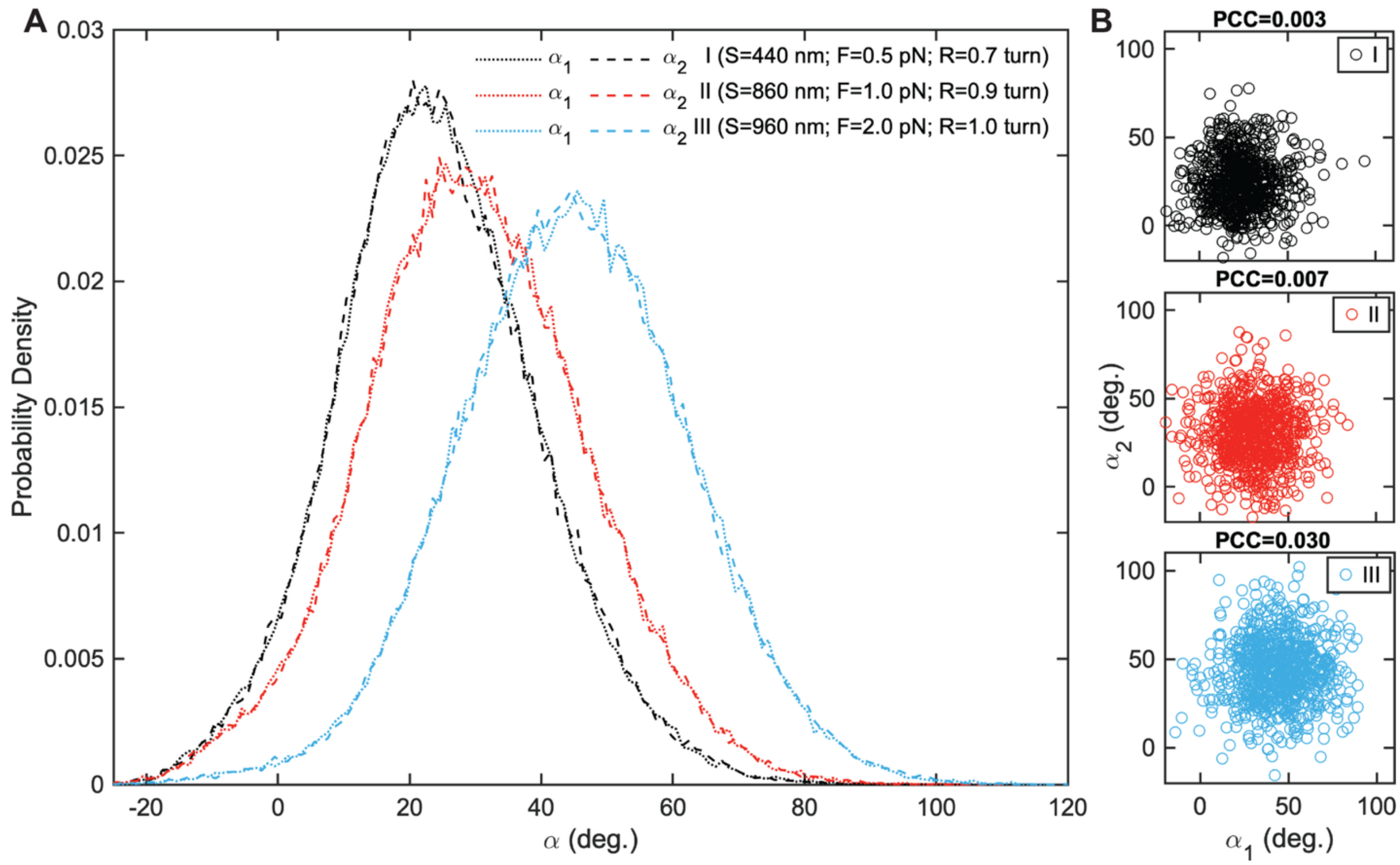

Figure S3: Similarity of representative probability distributions of α_1 and α_2 **A.** Probability density distributions of α_1 (short dashed lines) and α_2 (long dashed lines) at three different levels of Hookedness. **B.** Scatter plots of α_2 as a function of α_1 at three different levels of Hookedness. Each dot represents α for every 100th timestep from a simulation for a given configuration. The Pearson correlation coefficient (PCC) is included at the top of each scatter plot. The histograms and scatter plots are color-coded for each tuple ({S, F, R} = {440 nm, 0.5 pN, 0.7 turn}: Black; {860 nm, 1 pN, 0.9 turn}: Red; {960 nm, 2 pN, 1 turn}: Blue).

bioRxiv preprint doi: https://doi.org/10.64898/2026.09.04.749274; this version posted September 8, 2026. The copyright holder for this preprint (which was not certified by peer review) is the author/funder. This article is a US Government work. It is not subject to copyright under 17 USC 105 and is also made available for use under a CC0 license.

S4. The mean values of crossing parameters at F = 0.5 pN

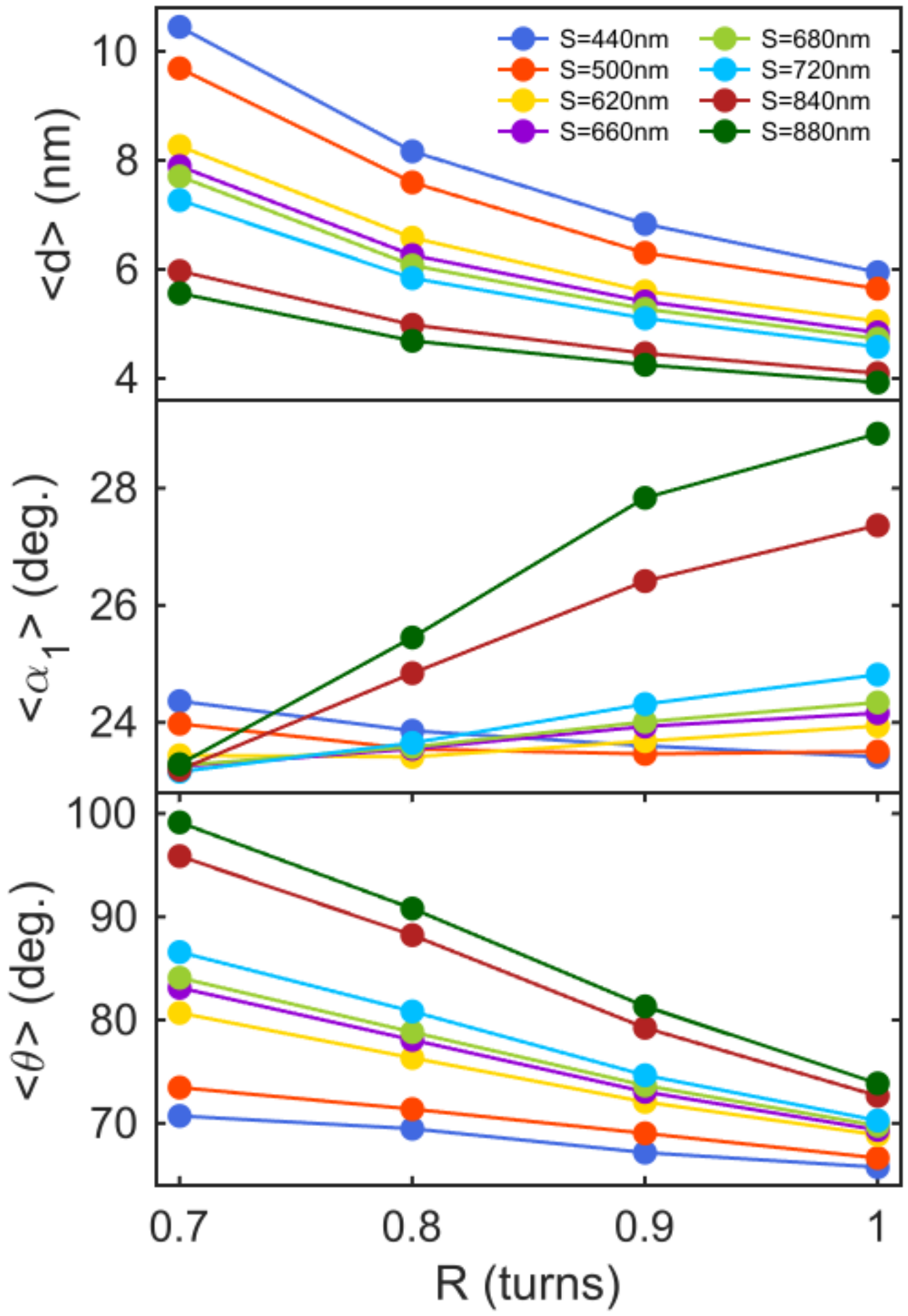

Figure S4: The mean values of crossing parameters, <*d*>, < $_1$>, < >, as a function of bead rotation (R) from simulations conducted at 0.5 pN force for different segment separation (S) values. The mean values from simulations performed at different segment separations are color-coded (S = 440 nm: Royal blue; S = 500nm: Orange red; 620 nm: Gold; 660 nm: Dark violet; 680 nm: Yellow green; 720 nm: Deep sky blue; 840 nm: Firebrick; 880 nm: Dark green).

bioRxiv preprint doi: https://doi.org/10.64898/2026.09.04.749274; this version posted September 8, 2026. The copyright holder for this preprint (which was not certified by peer review) is the author/funder. This article is a US Government work. It is not subject to copyright under 17 USC 105 and is also made available for use under a CC0 license.

S5. The mean values of crossing parameters at F = 1 pN

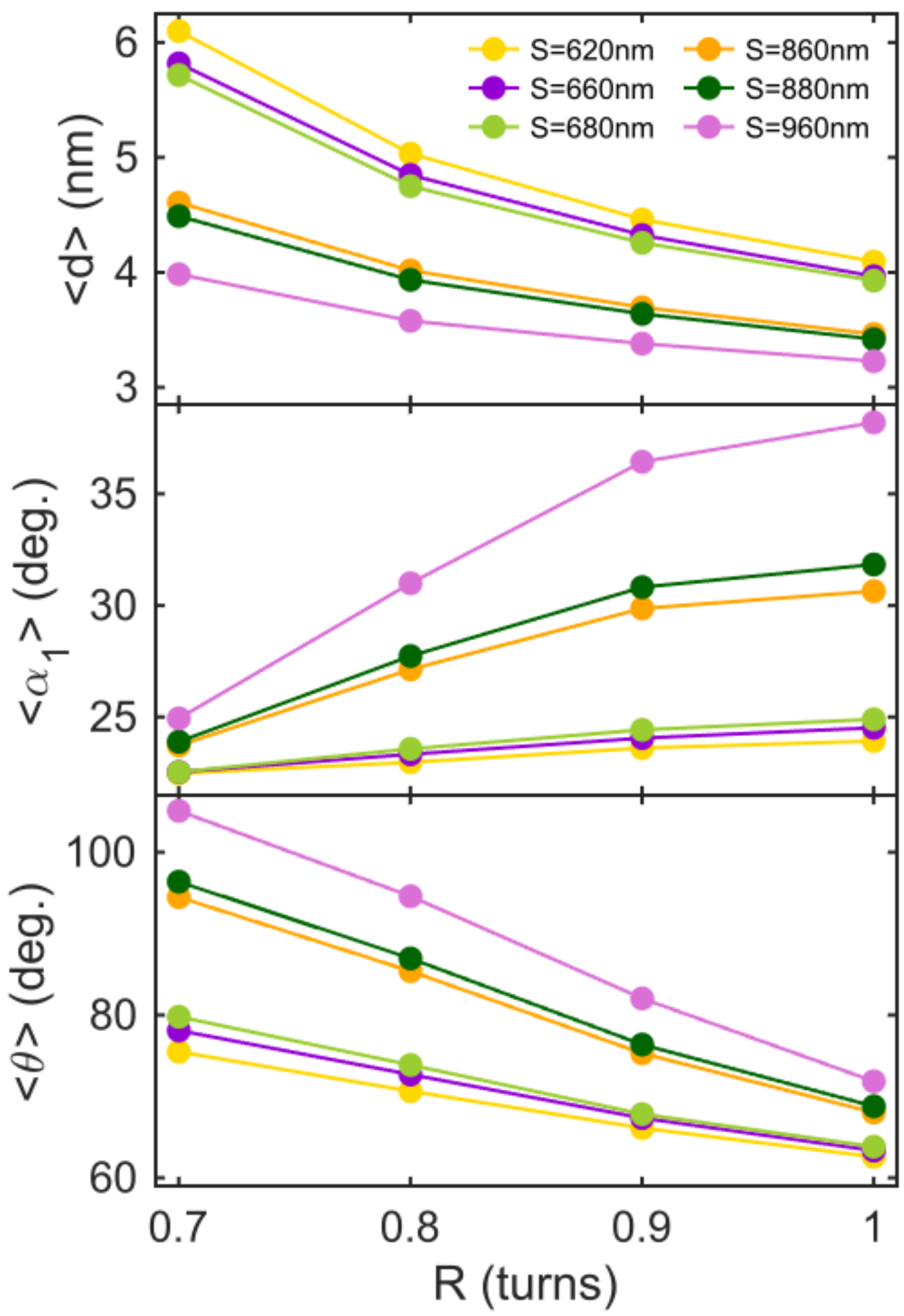

Figure S5: Mean values of geometric crossing parameters, <*d*>, < $_1$>, < >, as a function of bead rotation (R) from simulations conducted at F = 1 pN (S = 620nm: Gold; 660 nm: Dark violet; 680 nm: Yellow green; 860 nm: Orange; 880 nm: Dark green; 960 nm: Orchid).

bioRxiv preprint doi: https://doi.org/10.64898/2026.09.04.749274; this version posted September 8, 2026. The copyright holder for this preprint (which was not certified by peer review) is the author/funder. This article is a US Government work. It is not subject to copyright under 17 USC 105 and is also made available for use under a CC0 license.

S6. The mean values of crossing parameters at F = 2 pN

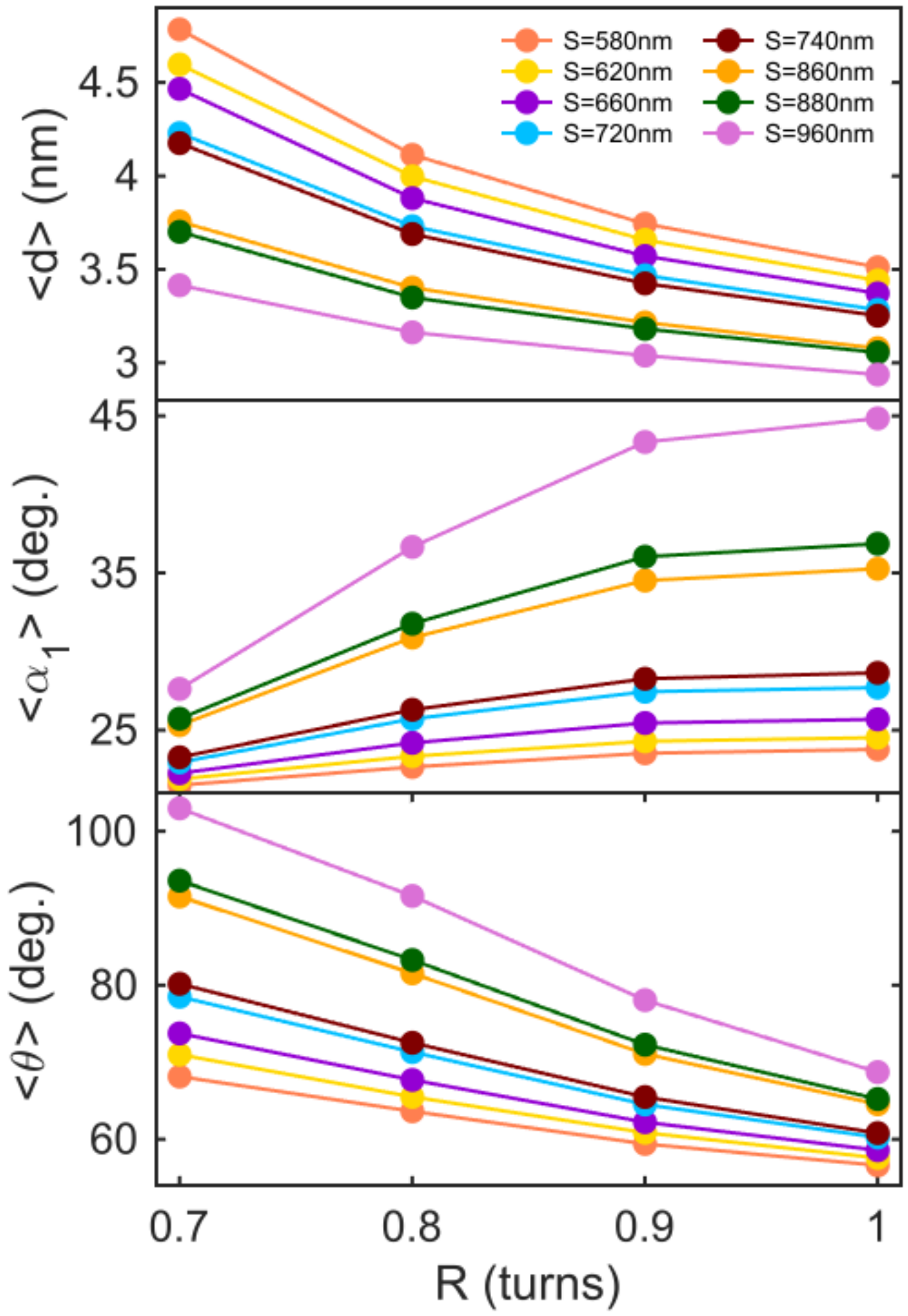

Figure S6: The mean values of crossing parameters, <*d*>, < $_{1}$>, < >, as a function of bead rotation (R) from simulations conducted at 2 pN force for different segment separation (S) values. The mean values from simulations performed at different segment separations are color-coded (S = 580nm: Coral; 620 nm: Gold; 660 nm: Dark violet; 720 nm: Deep sky blue; 740 nm: Maroon; 860 nm: Orange; 880 nm: Dark green; 960 nm: Orchid).

bioRxiv preprint doi: https://doi.org/10.64898/2026.09.04.749274; this version posted September 8, 2026. The copyright holder for this preprint (which was not certified by peer review) is the author/funder. This article is a US Government work. It is not subject to copyright under 17 USC 105 and is also made available for use under a CC0 license.

S7. Enzyme unlinking rate as a function of mean values of crossing parameters

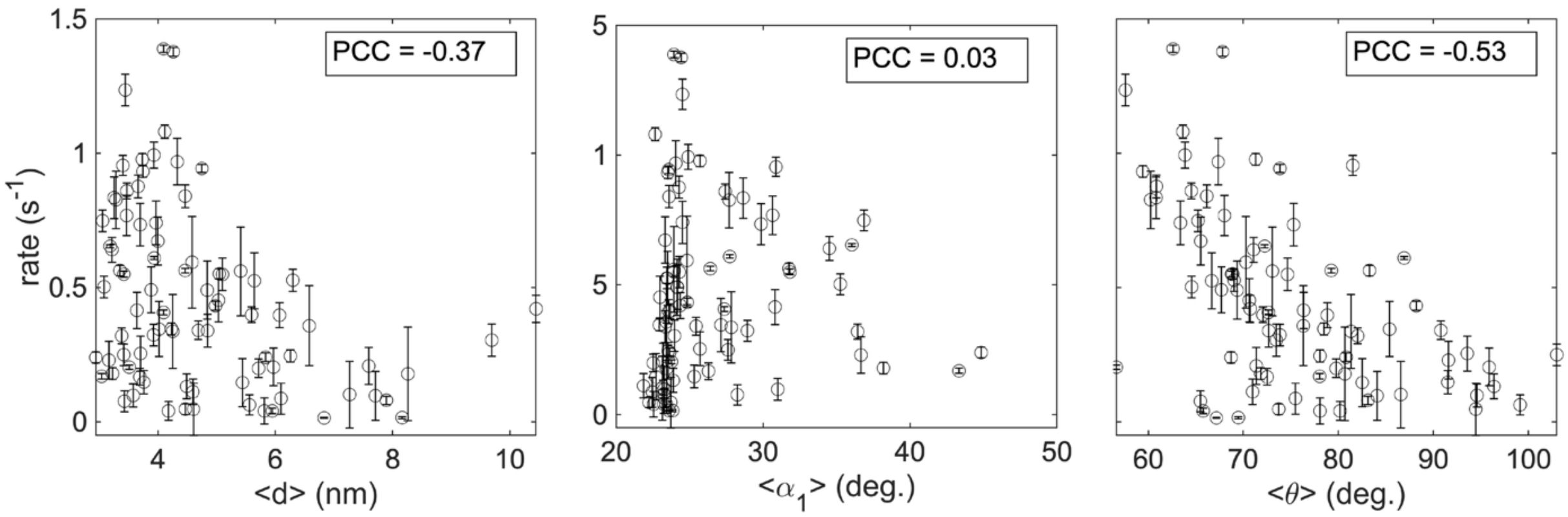

Figure S7: Scatter plot of enzyme unlinking rate as a function of computed mean values of crossing parameters, <*d*>, < α_1>, and <θ> along with the computed Pearson correlation coefficients (PCC).

bioRxiv preprint doi: https://doi.org/10.64898/2026.09.04.749274; this version posted September 8, 2026. The copyright holder for this preprint (which was not certified by peer review) is the author/funder. This article is a US Government work. It is not subject to copyright under 17 USC 105 and is also made available for use under a CC0 license.

S8. Enzyme unlinking rate as a function of computed joint probability for highest correlated geometric parameters

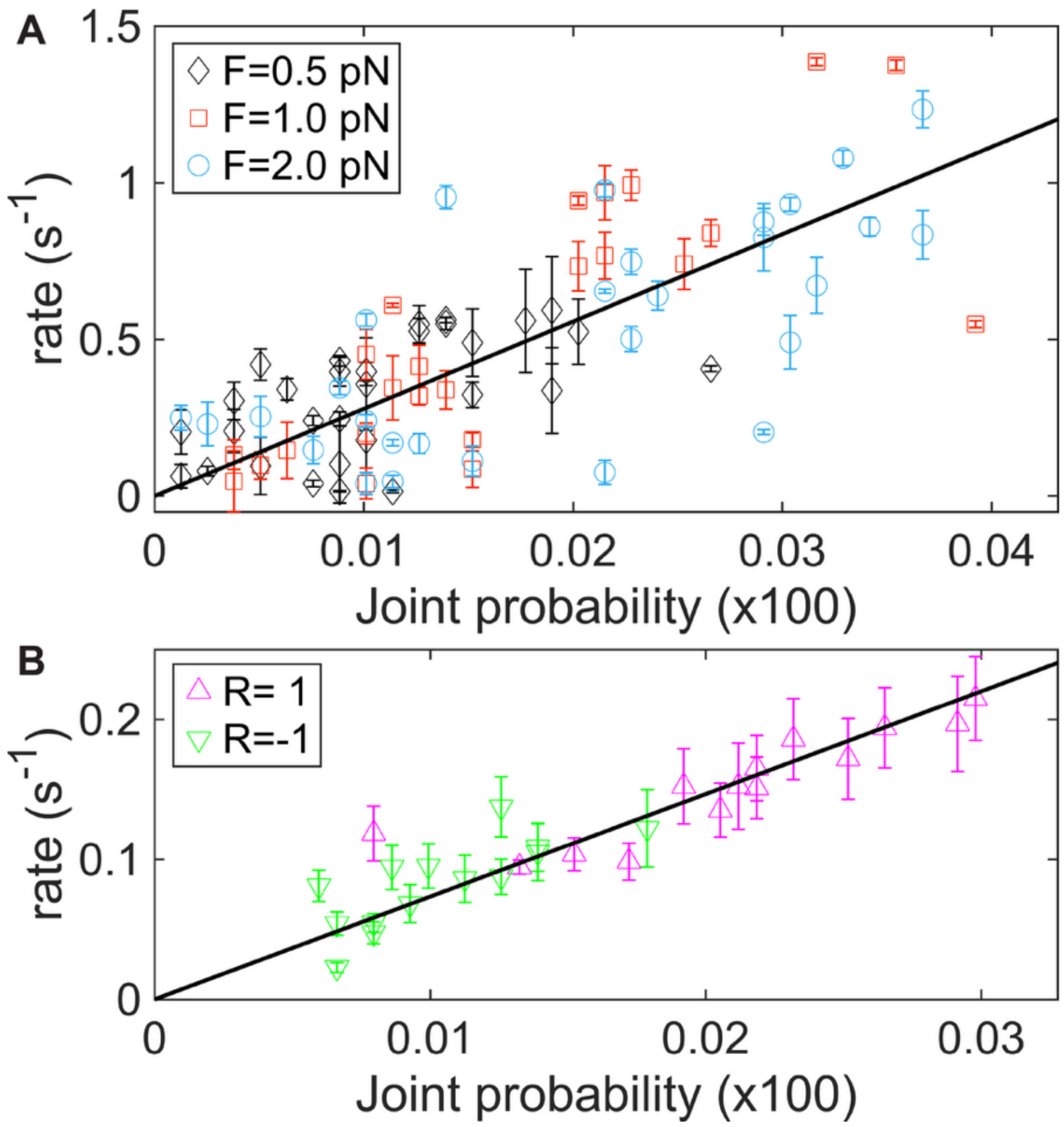

Figure S8: Scatter plots of enzyme unlinking rate as a function of computed joint probability with geometric parameters maximizing the correlation. **A.** Topo IV unlinking rate plotted as a function of the joint probability. The line shows a linear fit (slope = 27.9 ± 1.4 S^{-1}, with 0 intercept, and reduced chi-square, χ^2_υ = 460). To identify possible effects of force on the measured unlinking rate independent of the geometry, we computed the reduced chi-squared for the data at each force independently: $\chi^2_\upsilon|_{F=0.5\ pN}$ = 744, $\chi^2_\upsilon|_{F=1\ pN}$ = 340, $\chi^2_\upsilon|_{F=2\ pN}$ = 297. The decrease in the reduced chi-squared for with increasing force, and the lack of a systematic shift in the unlinking rates measured at different forces suggest that the rates do not decrease dramatically over the force range (0.5-2 pN). The unlinking rates for different forces are color- and symbol-coded (F=0.5 pN: Black diamond; 1 pN: Red rectangle; 2 pN: Blue circles). **B.** Topo VI unlinking rate (from previous work (1)) plotted as a function of the joint probability. The line shows a linear fit (with 0 intercept, slope = 9.4 ± 0.3 S^{-1}, and reduced chi-square, χ^2_υ = 2.1). The unlinking rates for different crossing signs (1 full positive or negative turn) are color-coded (R=1: Magenta upward triangle; R=-1: Green downward triangle). Error bars correspond to the standard error of the mean.

bioRxiv preprint doi: https://doi.org/10.64898/2026.09.04.749274; this version posted September 8, 2026. The copyright holder for this preprint (which was not certified by peer review) is the author/funder. This article is a US Government work. It is not subject to copyright under 17 USC 105 and is also made available for use under a CC0 license.

S9. Calculation of the collated crossing angle for all the pairs with a juxtaposition distance below a threshold value

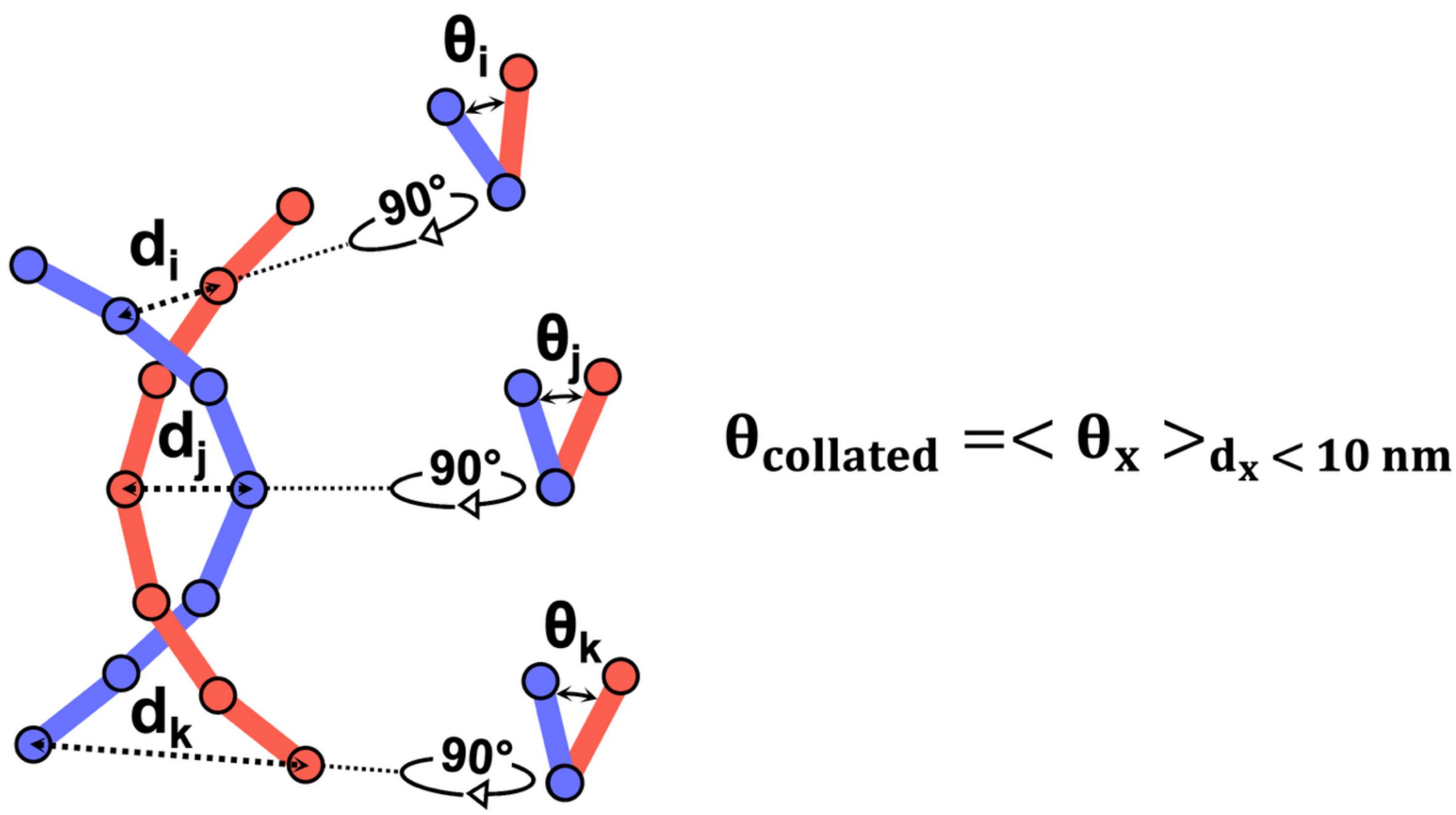

Figure S9: Schematic describing the calculation of collated DNA crossing angle in previous work (1, 2). To avoid confusion, we denote the crossing angle defined in prior work as   . At each simulation timestep, all possible crossings with a juxtaposition distance below a threshold value, 10 nm, were considered and the crossing angle was calculated for all such crossings (1, 2). The reported crossing angle (   ) in the previous work is the mean of individual crossing angle values measured at a given timestep (1, 2).

bioRxiv preprint doi: https://doi.org/10.64898/2026.09.04.749274; this version posted September 8, 2026. The copyright holder for this preprint (which was not certified by peer review) is the author/funder. This article is a US Government work. It is not subject to copyright under 17 USC 105 and is also made available for use under a CC0 license.

S10. Comparison of crossing geometries achieved in single DNA-crossings for different sizes of DNA

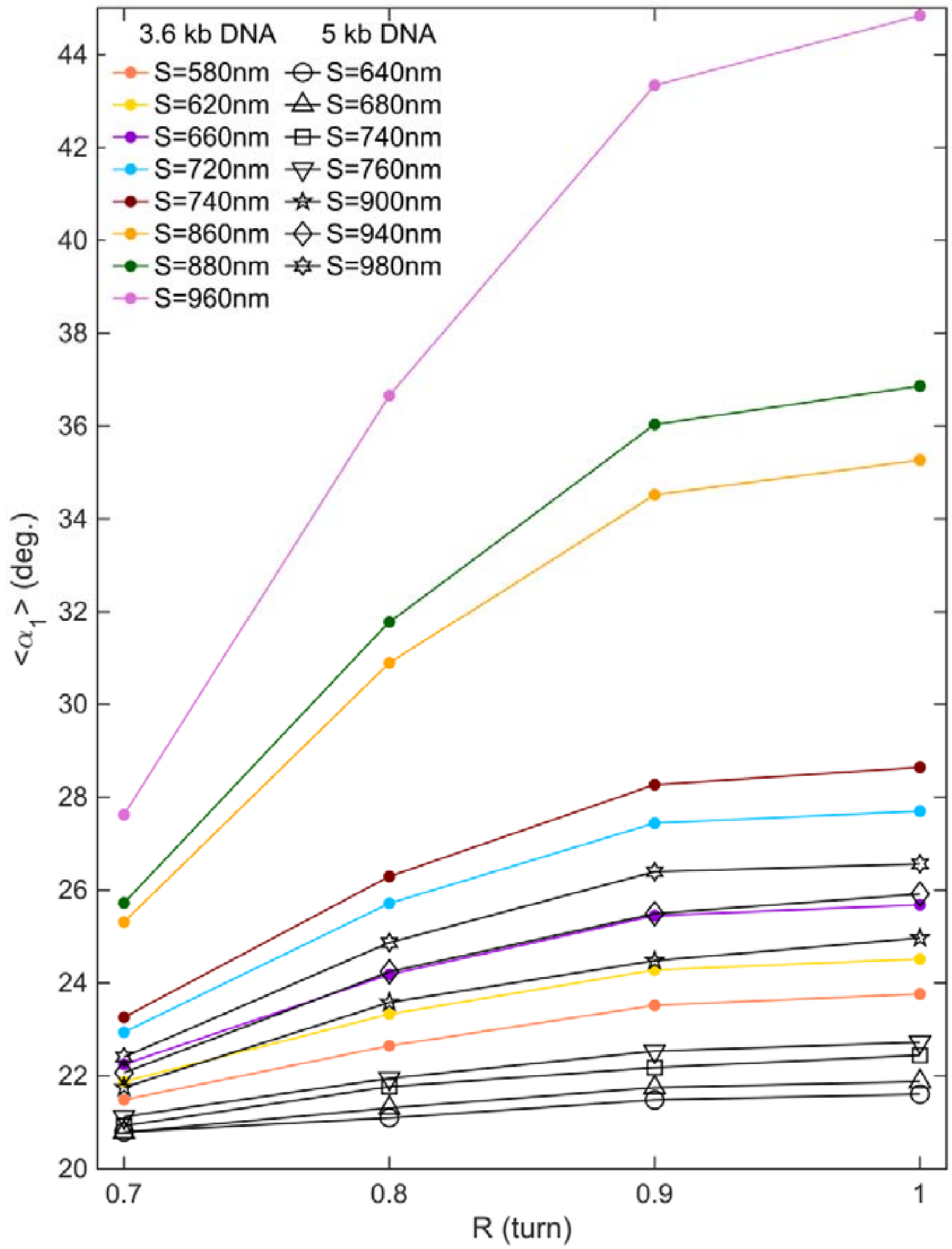

Figure S10: Comparison of crossing geometries achieved in single DNA-crossings for 3.6 kb and 5kb DNA. The mean values of bend angle, $<\alpha_1>$, are plotted as a function of bead rotation (R) from simulations conducted at 2 pN force for different segment separation (S) values. The mean values from simulations performed at different segment separations are color coded for 3.6kb DNA (S = 580nm: Coral; 620 nm: Gold; 660 nm: Dark violet; 720 nm: Deep sky blue; 740 nm: Maroon; 860 nm: Orange; 880 nm: Dark green; 960 nm: Orchid) and are symbol coded for 5kb DNA (S = 640nm: Circle; 680 nm: Upward-pointing triangle; 740 nm: Square; 760 nm: Downward-pointing triangle; 900 nm: Pentagram; 940 nm: Diamond; 980 nm: Hexagram). The shorter 3.6 kb DNA molecules resulted in a much larger range of bend angles and “hookedness” as a function of bead rotation than the 5 kb DNA molecules used in previous unlinking measurements. The large variation in the imposed bend angles permits a sensitive measure of the relation between bend angle and topo IV unlinking rate.

bioRxiv preprint doi: https://doi.org/10.64898/2026.09.04.749274; this version posted September 8, 2026. The copyright holder for this preprint (which was not certified by peer review) is the author/funder. This article is a US Government work. It is not subject to copyright under 17 USC 105 and is also made available for use under a CC0 license.

S11. Alternative analysis of simulations without parameterizing the crossings

The preferred crossings of topo IV obtained from the simulations were predicated on the geometric parameters defining the HJP model. To confirm our estimates of the preferred crossing geometry of topo IV, we performed an independent correlation analysis based on the pairwise distances between each bead in the pair of 5-bead arcs defining the crossing (Fig. S1). We considered the pairwise distance for 45 pairs in each crossing (Fig. S11). The correlation analysis of mean values of these pair-wise distances with unlinking rates gives a spectrum of correlation coefficients (Table S2). The strong anti-correlations (<-0.4) for pairs with non-central monomer beads are consistent with the weakly bent segments observed in the preferred crossing. Similarly, the moderate correlations for the intra-segment pair of neighboring beads to the central bead and the loss of correlation for the intra-segment pair of terminal beads do not support a sharp symmetric bend in the crossings. This approach offers an alternative, though qualitative, perspective on the preferred crossing geometry that is consistent with the preferred crossing geometry obtained through the correlation analysis.

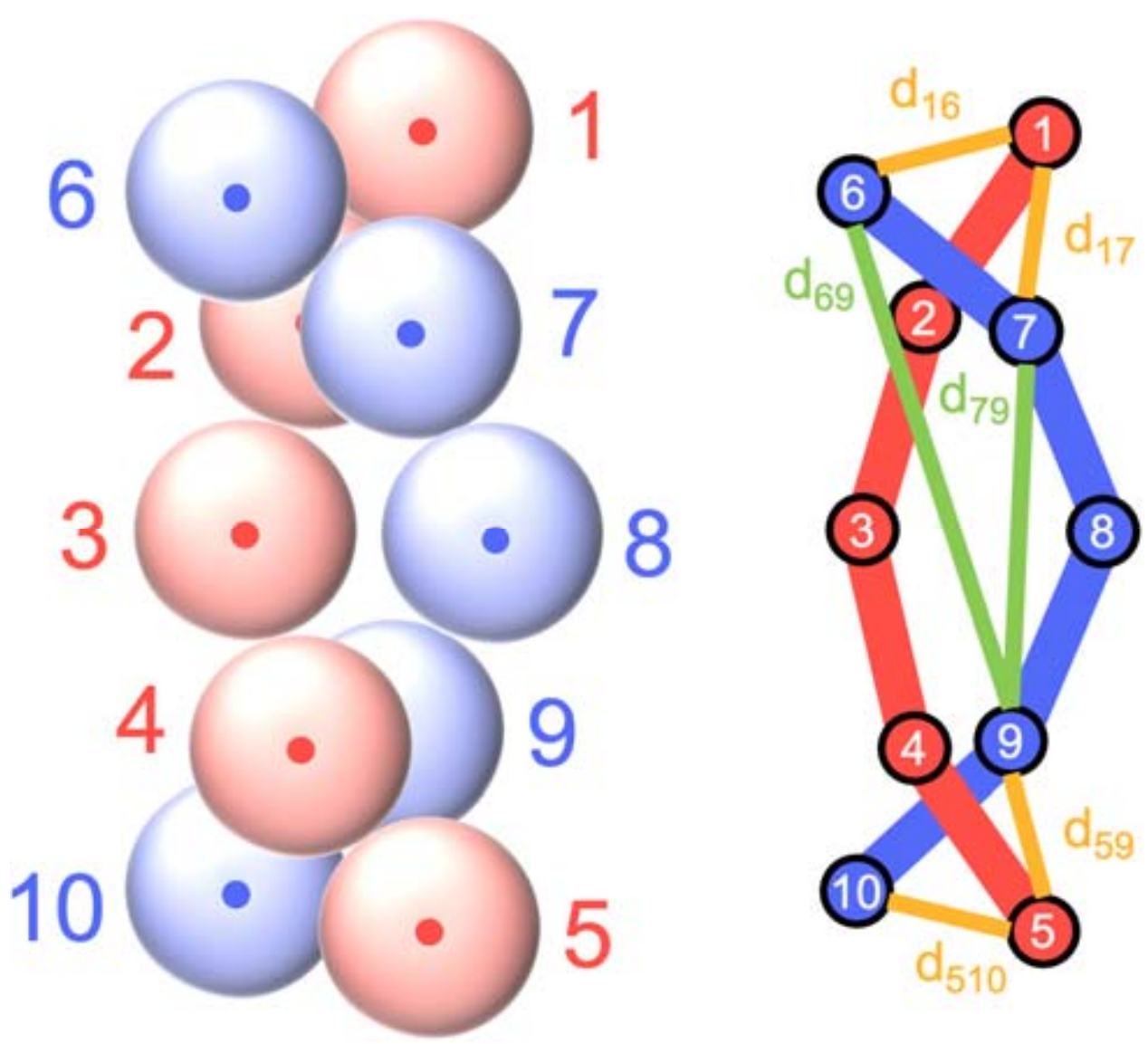

Figure S11: Schematic of a crossing of 5-monomer-bead segments from each dsDNA with the simplified ball-and-stick model (representing particle-and-bond) for easier visualization. The thin lines with labels show representative pair-wise distances.

bioRxiv preprint doi: https://doi.org/10.64898/2026.09.04.749274; this version posted September 8, 2026. The copyright holder for this preprint (which was not certified by peer review) is the author/funder. This article is a US Government work. It is not subject to copyright under 17 USC 105 and is also made available for use under a CC0 license.

S12. Comparison of DNA bending energy for different bend angles

The bending energy of a DNA segment bent through a radius can be expressed as:

$$U_{BEND,segment} = \frac{L_p L}{2r^2} k_B T$$

Where, L_p is the DNA persistence length, L is the DNA segment length, r is the radius of curvature of the segment, and $k_B T$ is the thermal energy. The bending energy can be expressed in terms of the bend angle (α) by replacing r with L/α in the above formula:

$$U_{BEND,segment} = \frac{L_p \alpha^2}{2L} k_B T$$

Hence, for a symmetric crossing with two uniformly bent segments,

$$U_{BEND,crossing} = \frac{L_p \alpha^2}{L} k_B T$$

We consider $L_p = 50\ \mathrm{nm}$, $L = 12.5\ \mathrm{nm}$ for further calculations

For the preferred crossing geometry ($\alpha = 37°$)

$$U_{BEND,crossing}\Big|_{\alpha=37°} = 1.7\ k_B T.$$

While, for the HJP crossing geometry ($\alpha = 165°$)

$$U_{BEND,crossing}\Big|_{\alpha=165°} = 33.2\ k_B T.$$

bioRxiv preprint doi: https://doi.org/10.64898/2026.09.04.749274; this version posted September 8, 2026. The copyright holder for this preprint (which was not certified by peer review) is the author/funder. This article is a US Government work. It is not subject to copyright under 17 USC 105 and is also made available for use under a CC0 license.

Table S1: Simulation parameters in the reduced Lennard–Jones (LJ) and SI units. The conversion of LJ units to SI units is not straightforward. Here, the LJ units are converted to SI units using the parameters provided on the LAMMPS website (https://docs.lammps.org/units.html).

	LJ units	SI units
Length	σ_{LJ}	2.5 nm
Time	τ_{LJ}	0.11 ns
Energy	$\square_{\mathrm{LJ}}$	4.14 pN nm
Force	F_{LJ}	0.61 pN

bioRxiv preprint doi: https://doi.org/10.64898/2026.09.04.749274; this version posted September 8, 2026. The copyright holder for this preprint (which was not certified by peer review) is the author/funder. This article is a US Government work. It is not subject to copyright under 17 USC 105 and is also made available for use under a CC0 license.

Table S2: Correlation coefficients between unlinking rates and the mean values of different pair-wise distances in crossings. Column 1 some representative categories (color codes as Inter-segment pair: Orange; Intra-segment pair: Green) of the pairs shown in Columns 2 and 3 (color codes as Monomer from the first segment: Red; Monomer from the second segment: Blue). Columns 4 shows the Pearson correlation coefficient for corresponding unlinking rates. Columns 5 shows the associated p-values for the correlation calculations. The table shows results for 45 pairs in the ascending order of correlation coefficients.

Pair type	ID_1	ID_2	PCC	p_value
terminal	1	6	-0.50	0.0000
terminal	5	10	-0.50	0.0000
	1	7	-0.45	0.0000
	4	10	-0.45	0.0000
	2	6	-0.45	0.0000
	5	9	-0.45	0.0000
	2	7	-0.42	0.0001
	4	9	-0.42	0.0001
central	3	8	-0.37	0.0006
	3	9	-0.36	0.0007
	2	8	-0.36	0.0007
	4	8	-0.36	0.0007
	3	7	-0.36	0.0007
central-terminal	1	8	-0.35	0.0010
central-terminal	3	10	-0.35	0.0010
central-terminal	5	8	-0.35	0.0010
central-terminal	3	6	-0.35	0.0010
	2	9	-0.30	0.0064
	4	7	-0.30	0.0066
	1	9	-0.24	0.0286
	2	10	-0.24	0.0287
	5	7	-0.24	0.0292
	4	6	-0.24	0.0292
trans terminal	1	10	-0.14	0.2078
trans terminal	5	6	-0.14	0.2120
	6	8	-0.06	0.5793
	8	10	-0.06	0.5853
	1	3	-0.06	0.6007
	3	5	-0.06	0.6121
terminal bend	1	5	0.01	0.9095
terminal bend	6	10	0.01	0.9089
	6	9	0.06	0.5979
	2	5	0.06	0.5775
	1	4	0.06	0.5701
	7	10	0.06	0.5675
bond	4	5	0.08	0.4893
bond	6	7	0.08	0.4612
bond	8	9	0.10	0.3914
bond	1	2	0.10	0.3818
bond	9	10	0.11	0.3241
bond	7	8	0.12	0.2943
bond	2	3	0.13	0.2387
bond	3	4	0.14	0.2128
mid bend	7	9	0.28	0.0117
mid bend	2	4	0.29	0.0083

Monomer ID of segment 1

Monomer ID of segment 2

Linearized crossing with monomer IDs

1	6
2	7
3	8
4	9
5	10

Pair type

Inter-segment pair

Intra-segment pair

bioRxiv preprint doi: https://doi.org/10.64898/2026.09.04.749274; this version posted September 8, 2026. The copyright holder for this preprint (which was not certified by peer review) is the author/funder. This article is a US Government work. It is not subject to copyright under 17 USC 105 and is also made available for use under a CC0 license.

Movie S1 (separate file). Representative simulations movie illustrating crossings and its thermal motion at different degrees of hookedness. Low hookedness crossing (**I**): S = 440 nm, F = 0.5 pN, R = 0.7 turn (252°). Medium hookedness crossing (**II**): S = 860 nm, F = 1 pN, R = 0.9 turn (324°). High hookedness crossing (**II**): S = 960 nm, F = 2 pN, R = 1 turn (360°).

SI References

1. S. J. McKie *et al.*, Topoisomerase V**I** is a chirally-selective, preferential DNA decatenase. *eLife* 11, e67021 (2022).

2. K. C. Neuman, G. Charvin, D. Bensimon, V. Croquette, Mechanisms of chiral discrimination by topoisomerase **I**V. *Proc. Natl. Acad. Sci. U.S.A.* 106, 6986-6991 (2009).
